# An Estimate of Plasma Volume Changes Following Moderate-High Intensity Running and Cycling Exercise and Adrenaline Infusion

**DOI:** 10.3389/fphys.2022.948087

**Published:** 2022-07-22

**Authors:** Jonathan J. Bjerre-Bastos, Casper Sejersen, Asger R. Bihlet, Niels H. Secher, Abigail L. Mackey, Carl-Christian Kitchen, Patryk Drobinski, Christian S. Thudium, Henning Bay Nielsen

**Affiliations:** ^1^ Xlab, Department of Biomedical Sciences, Faculty of Health and Medical Sciences, University of Copenhagen, Copenhagen, Denmark; ^2^ NBCD A/S, Herlev, Denmark; ^3^ Department of Anesthesia, Rigshospitalet, Department of Clinical Medicine, University of Copenhagen, Copenhagen, Denmark; ^4^ Institute of Sports Medicine, Bispebjerg Hospital, Department of Clinical Medicine, University of Copenhagen, Copenhagen, Denmark; ^5^ Nordic Bioscience, Herlev, Denmark; ^6^ Department of Anesthesiology, Roskilde Hospital, Department of Clinical Medicine, University of Copenhagen, Copenhagen, Denmark; ^7^ Department of Nutrition, Exercise and Sports, Faculty of Science, University of Copenhagen, Copenhagen, Denmark

**Keywords:** plasma volume change, biomarkers, adrenaline, exercise, running, cycling

## Abstract

**Introduction:** Plasma volume (PV) changes in response to physical activity, possibly as a consequence of adrenergic activation. We estimated changes in PV in response to common exercise modalities; cycling and running as well as adrenaline infusion and control at rest.

**Methods:** On separate days, forty circulatory healthy subjects [aged 60 years (range: 42–75)] with knee osteoarthritis underwent moderate-high intensity cycling, running, and intravenous adrenaline infusion to mimic the circulatory response to exercise. Blood samples were obtained from peripheral veins taken at several pre-defined time points before, during, and after the interventions. PV changes were estimated using venous hemoglobin and the derived hematocrit. The temporal associations between PV and selected biomarkers were explored.

**Results:** Changes in PV were observed during all four interventions, and the response to cycling and running was similar. Compared to rest, PV decreased by -14.3% (95% CI: -10.0 to -18.7) after cycling, -13.9% (95% CI: -10.9 to -17.0) after running, and -7.8% (95% CI: -4.2 to -11.5) after adrenaline infusion.

**Conclusion:** PV decreased in response to moderate-high intensity running and cycling. Adrenaline infusion mimicked the PV change observed during exercise, suggesting a separate influence of autonomic control on blood volume homeostasis. In perspective, a temporal association between PV and biomarker dynamics suggests that consideration of PV changes could be relevant when reporting plasma/serum constituents measured during exercise, but more research is needed to confirm this.

## Introduction

Plasma volume (PV) changes in response to physical activity, and thus it is relevant to take PV changes into consideration when investigating changes in plasma constituents. Plasma constituents, such as extracellular matrix (ECM) fragments or neoepitopes, act as biomarkers for osteoarthritis (OA) (van Spil and Szilagyi, 2020). Obtaining blood-based biochemical markers is minimally invasive and could reflect disease severity, disease activity, prognosis, and pharmacological target engagement. Several factors influence analysis results for biochemical markers, which are often plasma constituents sensitive to physiological changes prior to sampling as well as sample handling (Bjerre-Bastos et al., 2020). An important physiological factor is changes in PV in response to physical activity including exercise, the activity of daily living, and changes in posture (Johansen et al., 1998). Thus, it seems reasonable to consider adjustment for potential PV fluctuations when investigating plasma constituents, e.g., during or shortly following exercise, as in studies of acute joint tissue turnover (Roberts et al., 2019; Bjerre-Bastos et al., 2020).

Changes in PV may be evaluated by different methods. Simple methods include the hemoglobin/hematocrit method (van Beaumont et al., 1973) or the calcium method (Alis et al., 2015). Alternatively, e.g., “Evans blue” is injected and its optical properties are utilized to determine PV changes using spectrophotometry (Gibson and Evans, 1937). In studies of acute PV change in response to exercise, the magnitude depended on the intensity, and changes were prompt and small to moderate (Kaltreider and Meneely, 1940; Novosadová, 1977; Bloomer and Farney, 2013). Although acute hemoconcentration is a well-described physiological response to exercise known from studies of predominantly young healthy males performing ergometer cycling, several questions remain undescribed including 1) is there an inherent effect of adrenergic circulatory stress on PV? 2) do PV changes depend on the weight-bearing element of exercise? and 3) to which degree does hemoconcentration occur in a population representing that of common joint disease studies? middle-aged to senior, predominantly females, and often obese patients with comorbidity.

In the current study, we estimated changes in plasma volume in relation to common exercise modalities; cycling and running and included a control intervention; passive cardiovascular exercise simulation by means of adrenaline infusion.

## Methods

**FIGURE 1:**
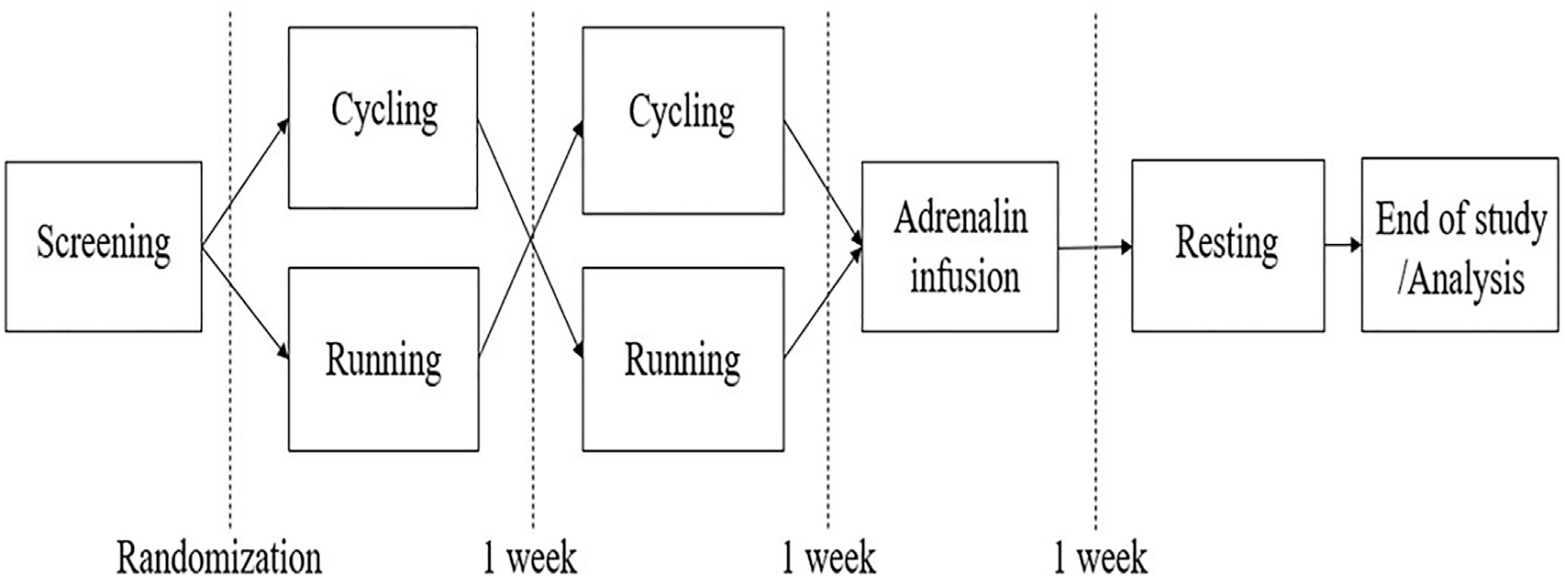
Study design.

The data presented in this communication originate from the EFEX-OA-02 exploratory clinical study (Bjerre-Bastos J. J. et al., 2022) in which the acute effect of exercise on biochemical markers reflecting joint tissue turnover in knee OA is investigated. The trial was based on the pilot study, EFEX-OA-01, as previously reported (Bjerre-Bastos et al., 2021a; 2021b), and the design is outlined in Figure 1. In EFEX-OA-02, hemoglobin and derived hematocrit were measured to monitor changes as a reflection of rapid fluctuations in PV which could bias the joint biomarker results.

Middle-aged and senior individuals with radiographic OA of the knee(s) with a cumulated Kellgren–Lawrence (KL) radiological grade of at least 2 and with no issues relating to the circulation were enrolled. Thus, the included patients had at least KL1 in both the knees or KL2 in one, were 20–75 years of age, weighed 50–100 kg, and had a BMI of 18.5–35 kg/m^2^. Exclusion criteria were knee OA KL grade 4 in one or both knees, cumulated osteoarthritis no higher than 1, history of arthroscopy or intra-articular injections in the knee within 6 months prior to screening, previous arthroplasty of knee or hip, intention to have major surgery during the timeframe of the study, secondary OA, e.g., related to trauma or inflammatory joint disease including chondrocalcinosis, treatment with beta-blockers, MAO inhibitors, systemic corticosteroid, vitamin K antagonists, new oral anticoagulants or heparin, systemic infection, systemic inflammatory immune, or autoimmune disease, and any sign of previous or current cardiovascular disease or being an active athlete or highly trained individual. The study was conducted in accordance with the standards of the Helsinki Declaration, approved by the Regional Ethical Committee (approval no. H-20026057), and registered at clinicaltrials.gov (reg. no. NCT04542668).

Participants underwent ergometer cycling (Ergomedic 874E, Monark Exercise AB, Varberg, Sweden) and treadmill running (Masterfit TP100, Abilica, Mylna Sport AS, Mjøndalen, Norway) in randomized order and then completed intravenous adrenaline infusion to mimic the circulatory load during exercise and finally a resting visit, with all interventions performed 1 week apart. Exercise and infusion bouts were multiphasic and included four progressive 5-min intervals. The maximal heart rate (HR_max_) was calculated as HR_max_ = 206.3-(0.711*age) (Londeree and Moeschberger, 1982). The resting heart rate (RHR) was determined experimentally at the screening by pulse oximetry during a 5-min supine rest. The heart rate reserve (HRR) was then calculated by the formula: HRR = HR_max_ – RHR. The targeted peak intensity during exercise interventions was >80% of the HRR.

At each intervention, blood samples were obtained at pre-defined time points before, during, and after the intervention. Participants were instructed to be minimally active on the day before and on the days of interventions. Participants were also instructed to fast for at least 6 hours prior to visits and throughout the study day until all samples had been obtained. All visits were initiated with a 30-min resting period in order to reduce potential signals from pre-visit physical activity such as stair-climbing, walking, etc. The outline of the study design is shown in Figure 1.

HR during exercise was monitored with a Polar™ H10 chest sensor belt (Kempele, Finland). For exercise simulation, 0.06 mg/kg of adrenaline was prepared in a 50 ml saline solution and administered intravenously using an Infusomat^®^ Space^®^ pump (B. Braun, Melsungen, Germany) with the participants semi-recumbent under continuous electrocardiographic monitoring by the study physician, adjusting the flow rate to mimic exercise intervals evaluated by the HR, while only inducing tolerable, if any, discomfort. Hemoglobin was measured on an ABL 800 Flex blood gas analyzer (Radiometer, Copenhagen, Denmark). Hemoglobin (ctHb) was converted to hematocrit by the formula:

Hct (%) = (0.0485 × ctHb (mmol/L) + 0.0083) × 100 (Kokholm, 1990).

The estimated change in PV (% ΔPV) was calculated as:

$\text{\%}\Delta \text{PV}=\left[\text{100/}\left(\text{100}-{\text{Hct}}_{\text{pre}}\right)\right]\text{x}\left[\text{100}\left({\text{Hct}}_{\text{pre}}-{\text{Hct}}_{\text{post}}\right){\text{/Hct}}_{\text{post}}\right]$ (van Beaumont et al., 1973),

where ΔPV % = percentage change in plasma volume, Hct_pre_ = hematocrit before exercise, and Hct_post_ = hematocrit after exercise.

On days of active intervention (exercise or infusion), blood was drawn at baseline (T_0_), once midway during exercise/infusion (T_10_), immediately after completion of the intervention (T_20_) and again at 30, 60, 120 and 240 min after T_20_ and on the next day approximately 24 h after the initiation of intervention. At rest, samples were collected at similar time points, except for the 24-h follow-up sample, which was omitted. Samples were collected from sitting participants during and after exercise and on the day of rest. During adrenaline infusion, the first three samples (during the interventions) were collected with the participant semi-recumbent, while the additional samples were collected from sitting participants.

Repeated measures of hemoglobin during running, cycling, infusion, and resting visits were tested for normality using Shapiro–Wilk’s test. A paired linear mixed-effects model with Dunnett’s multiple comparison test was used to assess changes in hemoglobin over time and compare interventions, and a multiple linear regression model with backward elimination was used to identify correlations between baseline subject characteristics and changes in hemoglobin from baseline, adjusting for age, sex, BMI, and knee pain.

The PV changes presented in the current report were subtracted from relative biomarker changes from the EFEX-OA-02 and compared to unadjusted changes in biomarkers in order to demonstrate the potential impact of adjustment for PV fluctuation.

Missing values were imputed with the average value of the previous and following values measured. Missing pre-intervention (baseline) values were imputed using an average of the pre-intervention values from the three other visits. Statistical analyses were carried out using GraphPad Prism 9.1.0 and MedCalc v. 20.009. *p* < 0.05 was considered significant.

## Results

Forty subjects met the inclusion criteria and none of the exclusion criteria of which 39 completed cycling, 37 completed running, 35 adrenaline infusion, and 39 rest. Sixteen (40%) were male, the mean age was 60.0 years (range: 42–75), BMI was 27.0 (standard deviation (SD): 3.5), baseline hemoglobin was 9.1 mmol/L (SD: 0.7), and hematocrit was 44.7% (SD: 2.9%). All subjects reached the minimum peak intensity or higher during exercise, while no participant reached the minimum peak intensity or higher during adrenaline infusion, with an average of 70.0% (SD: 8.7) of that during infusion. Twelve out of 1,151 sample values were imputed. None were baseline values, and all twelve were missing at random due to either loss of sample or sample error during ABL machine analysis.

**FIGURE 2:**
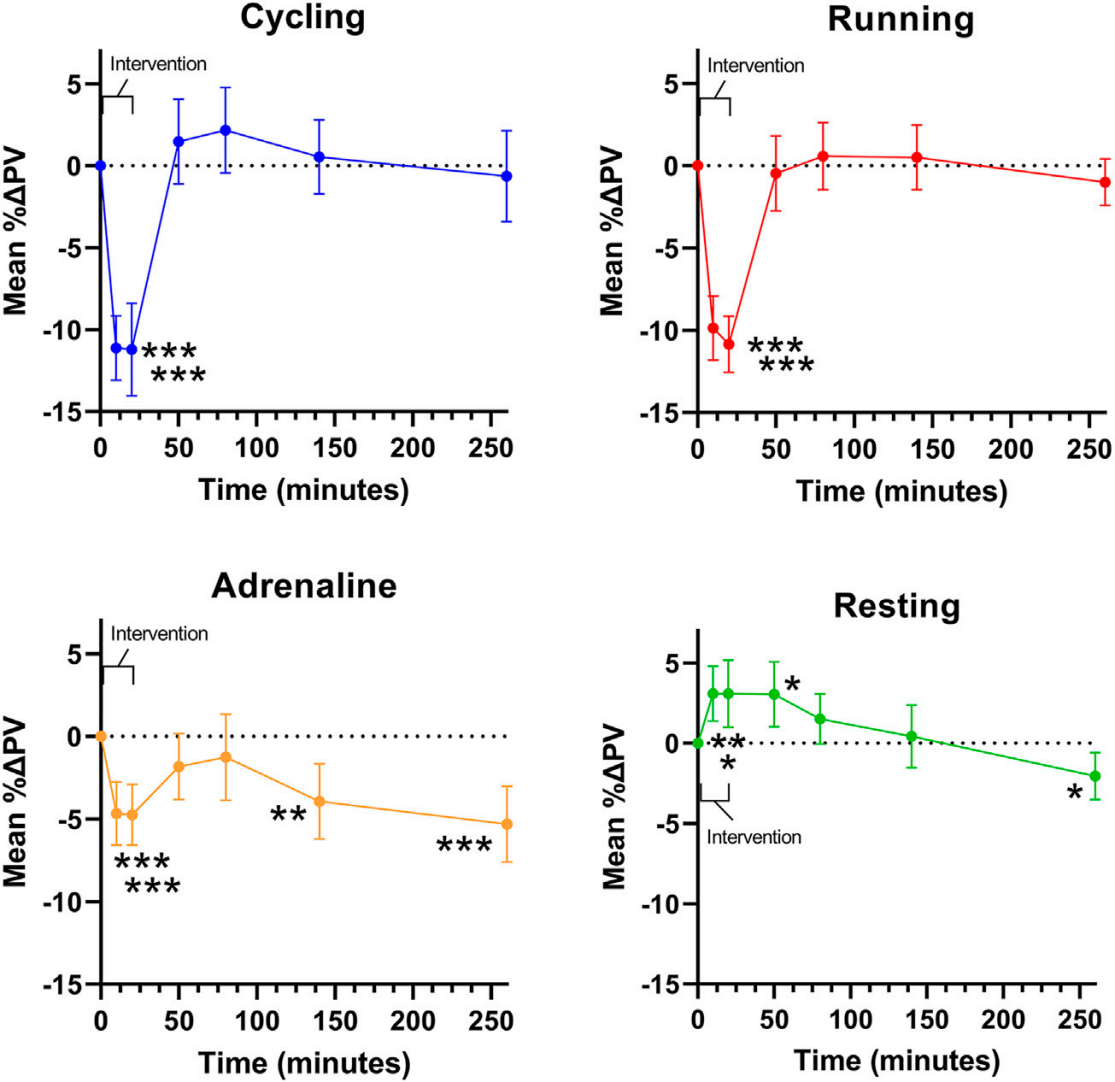
Mean changes in plasma volume (PV) over time during and following interventions. Error bars represent a 95% confidence interval.

Comparing PV changes immediately before (T_0_) and after interventions (T_20_), the mean PV reduction was -11.2% (95% CI: -7.2 to -15.6%) after cycling ranging from -17.0% increase to -24.0%. In one subject, PV did not change, and in five patients, PV increased by 2.0–4.0%. After running, the mean change was -10.8% (95% CI: 8.4 to -13.3%) ranging from 0.0 to -22.0% (Figure 2). In one subject, no change was observed. After the infusion of adrenaline, the mean change was -4.7% (95% CI: -2.1 to -7.2%) ranging from an 8.0% increase to a 17.8% decrease. In four subjects, there was no change, and in three subjects, an increase in PV was detected in response to cycling or running, but among these subjects, the response was not consequent across the active interventions. Among fourteen subjects with a substantial decrease in PV (>10%) after cycling or running, only three responded quantitatively consistently, although the other eleven also decreased.

Thirty minutes after exercise (T_50_), the mean PV had returned to the baseline level, and the day after both cycling and running, PV remained at the baseline level. PV returned to baseline value at 30–60 (T_50-80_) minutes after infusion but decreased further at T_140_ by -3.9% (95% CI: -0.7 to -7.1%), at T_260_ by -5.3% (95% CI: -2.1 to -8.6%), and on the next day by -5.9% (95% CI: -2.0 to -9.9%) compared to baseline. During seated rest, PV increased (3.1%, 95% CI: 0.7–5.4%) but returned to baseline and then slightly decreased (-2.0%, 95% CI: 0.0 to -4.1%) at T_260_. Thus, when comparing PV changes immediately after active interventions (cycling, running, adrenaline) to the corresponding resting sample, significant reductions in PV were detected as the difference was cycling -14.3% (95% CI: -10.0 to -18.7), running -13.9% (95% CI: -10.9 to -17.0), and adrenaline -7.8% (95% CI: -4.2 to -11.5) with the changes following exercise being significantly higher compared to those manifesting during the infusion (*p* < 0.001; Figure 2). There was no difference in the changes observed after cycling vs. running (*p* = 0.99) (Figure 2).

**FIGURE 3:**
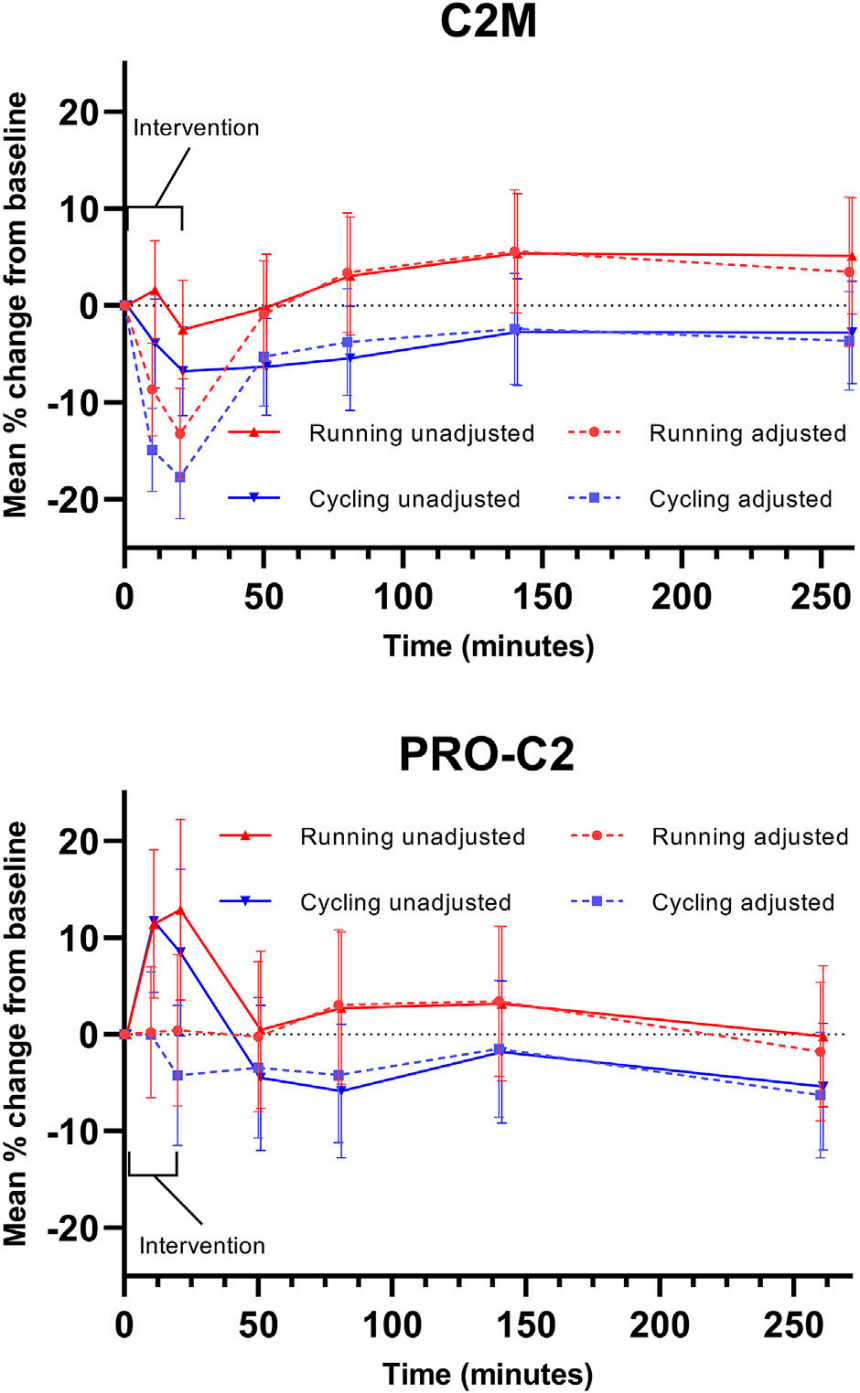
Adjustment in biochemical markers. Serum markers of type II collagen degradation (C2M) and repair (PRO-C2) are displayed. Error bars represent a 95% confidence interval.

Considering unadjusted changes, the biomarker of type II collagen degradation, C2M, was stable to the impact of exercise, while the biomarker of type II collagen formation, PRO-C2, increased during and immediately after exercise to return to baseline 30 min after. Adjusting for concurrent plasma volume fluctuation resulted in a significant decrease in C2M during and immediately following exercise, while adjustment in PRO-C2 eliminated all significant changes. (Figure 3).

Results of linear multiple regression adjusted for age, sex, BMI, and pain score demonstrated a borderline significant correlation between age (r = 0.32, *p* = 0.06), and the change in PV after running, suggesting that higher age was associated with less reduction in PV. Sex (r = -0.41, *p* = 0.01) correlated negatively with the change in PV after running, suggesting that male sex was associated with a higher reduction in PV.

## Discussion

This study investigated acute and subacute changes in plasma volume in relation to moderate-high intensity running, cycling, and intravenous adrenaline infusion as well as rest in circulatory healthy middle-aged and elderly subjects with knee osteoarthritis. The adrenaline intervention allowed for exploration of the inherent effects of circulatory stress without joint and muscle use or postural effects. Two major findings are reported: 1) cycling and running lead to similar reductions in PV and 2) infusion of adrenaline mimicked the PV change observed in response to exercise.

Thus, PV fluctuations were evaluated during multiple interventions in a middle-aged to senior and mixed-gender population. The aimed intensity was set to a realistic minimum of 80% of the HRR considering the potential limited exercise performance of the subjects due to OA and age. The intensity was reached for all participants and the achievements and magnitude of the associated PV change seem logical considering the population characteristics, and the results are even in line with PV changes reported for young, healthy males (Kaltreider and Meneely, 1940). The similar changes following cycling and running suggest that hemoconcentration appears to depend on the circulatory load rather than on the weight-bearing load. This is supported by the observation that the PV changed following adrenaline infusion. The circulatory load, reflected in HR, reached by means of adrenaline infusion was somewhat lower than that reached during exercise likely explaining the reduced impact on plasma volume of adrenaline infusion compared to exercise. In addition, as the infusion was performed when the subjects were semi-recumbent, there was likely a postural bias, counteracting hemoconcentration (Hinghofer-Szalkay and Moser, 1986). Our finding is in line with previous studies, where plasma volume was found to decrease by 7.6% (Hilsted et al., 1989) and hemoglobin was found to increase (Rokamp et al., 2017) in response to adrenaline infusion. The slight initial decrease in hemoglobin at rest corresponds to the recruitment of fluid from the legs (Hagan et al., 1978; Matzen et al., 1991) in previously upright humans. In a few subjects, we observed a negative or no change in hemoglobin in response to exercise, suggesting euvolemia and hemodilution, respectively. The regression analysis suggests that factors such as sex and age affect the PV change after running but not cycling and adrenaline infusion. Our regression analysis findings may therefore be explained by a difference in the response to weight-bearing work as compared to non-weight-bearing work. However, considering the limited sample size and power loss due to multiple covariates, this finding should be interpreted with caution.

Rapid PV changes were seen in an intensity dose-response manner following brief ergometer cycling (Kaltreider and Meneely, 1940). Hour-long aerobic ergometer cycling induced an 11% reduction in PV, while 15 s sprints induced a 19% reduction in young males (Bloomer and Farney, 2013). In young healthy females, 30 s of all-out pedaling induced a 10–15% reduction in PV (Rotstein et al., 1982). Novasodova (1977) tested the effect of three intensities of cycling in young healthy males: prolonged exercise at 40 and 67% as well as a session at 100% intensity. PV decreased 13% after maximal exercise, while hemoglobin increased 8.9%. A slightly smaller (5.9%) reduction was observed after the 67% intensity session, and no changes were observed after exercise at 40% intensity. Further, a linear relationship between hematocrit and hemoglobin was reported (Novosadová, 1977), supporting the methods chosen in the current report. In sedentary young males, a 5.3% increase in hemoglobin was observed after four 30-s Wingate tests (Belviranli et al., 2017). Older individuals may have altered function compared to young individuals such as increased leakage of fluid into the interstitial space or reduced hemoconcentration due to reduced homeostatic capacity. We investigated middle-aged to senior males and females with knee OA and no known or observed cardiovascular comorbidities. This population is different from previous studies and is similar to studies evaluating novel OA drugs. Thus, our findings indicate that the PV described for healthy young subjects in response to exercise also applies to middle-aged and elderly OA patients.

### Perspectives

In studies evaluating biochemical markers, it is important to know factors that may affect biomarker levels. Our results indicate slight to moderate hemoconcentration during and immediately after exercise that normalizes within 30 min. This study confirms Bloomer and Farney’s conclusion that plasma constituents measured in relation to exercise should be adjusted (Bloomer and Farney, 2013), and PV changes are taken into consideration when evaluating changes in plasma constituents in immediate response to exercise. Lack of adjustment for PV changes is also relevant for probably many, if not all, plasma constituents in response to exercise (Teixeira et al., 2014). Some plasma constituents may be redistributed from the bloodstream into the interstitial space during exercise, while other plasma constituents may stay in the concentrated circulation, and thus it may be necessary to be familiar with the kinetics of the relevant biomarker.

In Figure 3, the potential impact of adjustment for PV changes is illustrated using biomarker data from the EFEX-OA-02 study (Bjerre-Bastos J. et al., 2022; Bjerre-Bastos et al., 2022 J. J.). Two serum biomarkers are displayed: C2M, reflecting type II collagen degradation, and PRO-C2, reflecting type II collagen repair. As illustrated, the observed dynamics of biomarker PRO-C2 are temporally compatible with the observed reduction in PV in the acute phase where PV is reduced. Assuming concentration of the biomarker analyte in plasma as a result of PV reduction, adjustment for the reduced PV results in PRO-C2 levels which are not significantly different from baseline levels during this phase. Conversely, the observed changes in C2M are augmented by adjustment for reduced PV.

The tissue distribution kinetics of these two biomarkers in response to exercise-induced plasma redistribution is unknown, and thus the figures exemplify how considering PV changes may be relevant when measuring biochemical markers in direct relation to exercise, meaning during and immediately after. Lack of adjustment in samples obtained during or immediately after exercise may introduce a bias due to the shift in plasma volume, which could be misinterpreted as actual changes in the biochemical markers. In the current example, the adjustment in C2M turns the 10- and 20-min results into a significant change in the biomarker, while the initially observed PRO-C2 changes are virtually negated. The observed exercise-induced PV changes normalize and stabilize within 30 min after the bout, indicating that PV adjustment may have less relevance outside the acute window of exercise and on the long-term chronic effects of exercise on such markers. Past studies evaluating biochemical markers in an acute exercise setting generally did not adjust for acute plasma volume changes and observed prompt, transient increases in joint biochemical markers could therefore be biased (Andersson et al., 2006; Bjerre-Bastos et al., 2021a; Maren et al., 2022).

Measurement of changes in hemoglobin and hematocrit is a practically simple, cheap, and accessible way to evaluate plasma changes but is not considered as exact as more comprehensive methods (Johansen et al., 1998). The method of choice may depend on the expected magnitude of the changes in biochemical markers so that markers for which a substantial change may be expected are less relevant for PV adjustment and vice versa. Although methods based on hemoglobin/hematocrit may not be completely exact (Johansen et al., 1998), changes in hemoglobin have been shown to be in accord with PV changes (Kaltreider and Meneely, 1940) and are considered reliable. Another limitation is that intensity was not based on maximal oxygen uptake, but as this was a study of OA patients, pulse reserve was chosen based on the consensus recommendations for knee OA patients by the American Geriatric Society (Lundebjerg, 2001). Finally, subjects were fasted 6 hours prior to intervention/sample taking and throughout the 260-min sample periods, reducing nutritional bias. However, hydration status was not controlled, and this may limit the accuracy of our estimates.

## Conclusion

In middle-aged and elderly subjects, PV reduced ∼11% in response to moderate-high intensity running and cycling, suggesting that the circulatory stress is crucial and not the mechanical load. Moderate cardiovascular stress by means of intravenous adrenaline infusion mimicked PV reduction by ∼ 5%, suggesting a separate influence of autonomic control on blood volume homeostasis. In this perspective, the temporal associations with PV should probably be taken into account when addressing biochemical marker dynamics during exercise.

## Data Availability

The original contributions presented in the study are included in the article; further inquiries can be directed to the corresponding author.
